# Implementation of Clinical Practice Guidelines for Hospitalized Patients With COVID-19 in Academic Medical Centers

**DOI:** 10.1001/jamanetworkopen.2022.5657

**Published:** 2022-04-04

**Authors:** Amy Chang Berger, Noa Simchoni, Andrew Auerbach, W. Michael Brode, Ethan Kuperman, Katie Raffel, Alan Kubey

**Affiliations:** 1 Division of Hospital Medicine, Department of Medicine, University of California, San Francisco; 2 Division of Hospital Medicine, Department of Internal Medicine, Dell Medical School at University of Texas at Austin, Austin; 3 Division of General Internal Medicine, Department of Internal Medicine, University of Iowa, Iowa City; 4 Division of Hospital Internal Medicine, Department of Internal Medicine, Denver Health, Denver, Colorado; 5 Division of Hospital Medicine, Department of Medicine, Thomas Jefferson University Hospital, Philadelphia, Pennsylvania; 6 Division of Hospital Internal Medicine, Department of Internal Medicine, Mayo Clinic, Rochester, Minnesota

## Abstract

This survey study assesses the rate at which US academic medical centers have adopted evidenced-based guidelines for the management of COVID-19 into practice.

## Introduction

COVID-19 management has evolved rapidly, creating challenges for implementation. This study was conducted to assess the fidelity with which academic medical centers (AMCs) adopted evidence into practice.

## Methods

This survey study was deemed exempt from review and informed consent by the University of California, San Francisco, because it does not involve human participants. Response rates were computed according to the American Association for Public Opinion Research (AAPOR) reporting guideline.

**Figure 1.**
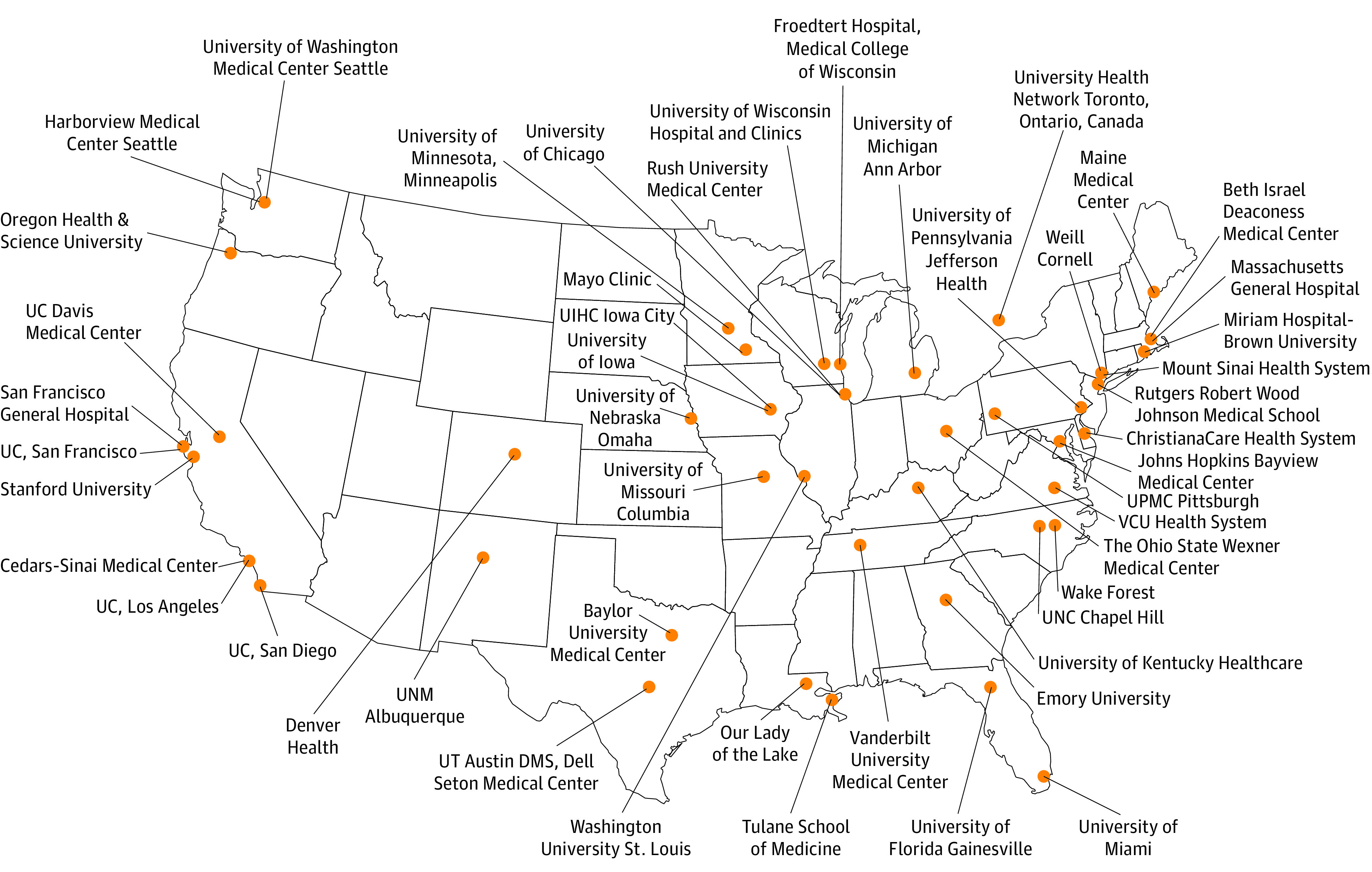
Map of Hospitals Participating in the Hospital Medicine Reengineering Network UC indicates University of California; UIHC, University of Iowa Hospitals and Clinics; UNC, University of North Carolina; UNM, University of New Mexico; UPMC, University of Pittsburgh Medical Center; UT, University of Texas; and VCU, Virginia Commonwealth University.

We surveyed members of the Hospital Medicine Reengineering Network (Figure 1)^1^ from December 17, 2020, to February 10, 2021, and compared their institutional recommendations for COVID-19 management with available evidence at that time from pivotal randomized clinical trials (RCTs) (eAppendix in the Supplement) and guidelines from the National Institutes of Health,^2^ Infectious Diseases Society of America,^3^ and American Society of Hematology.^4^ Data were analyzed from February 10 to March 4, 2021.

## Results

**Figure 2.**
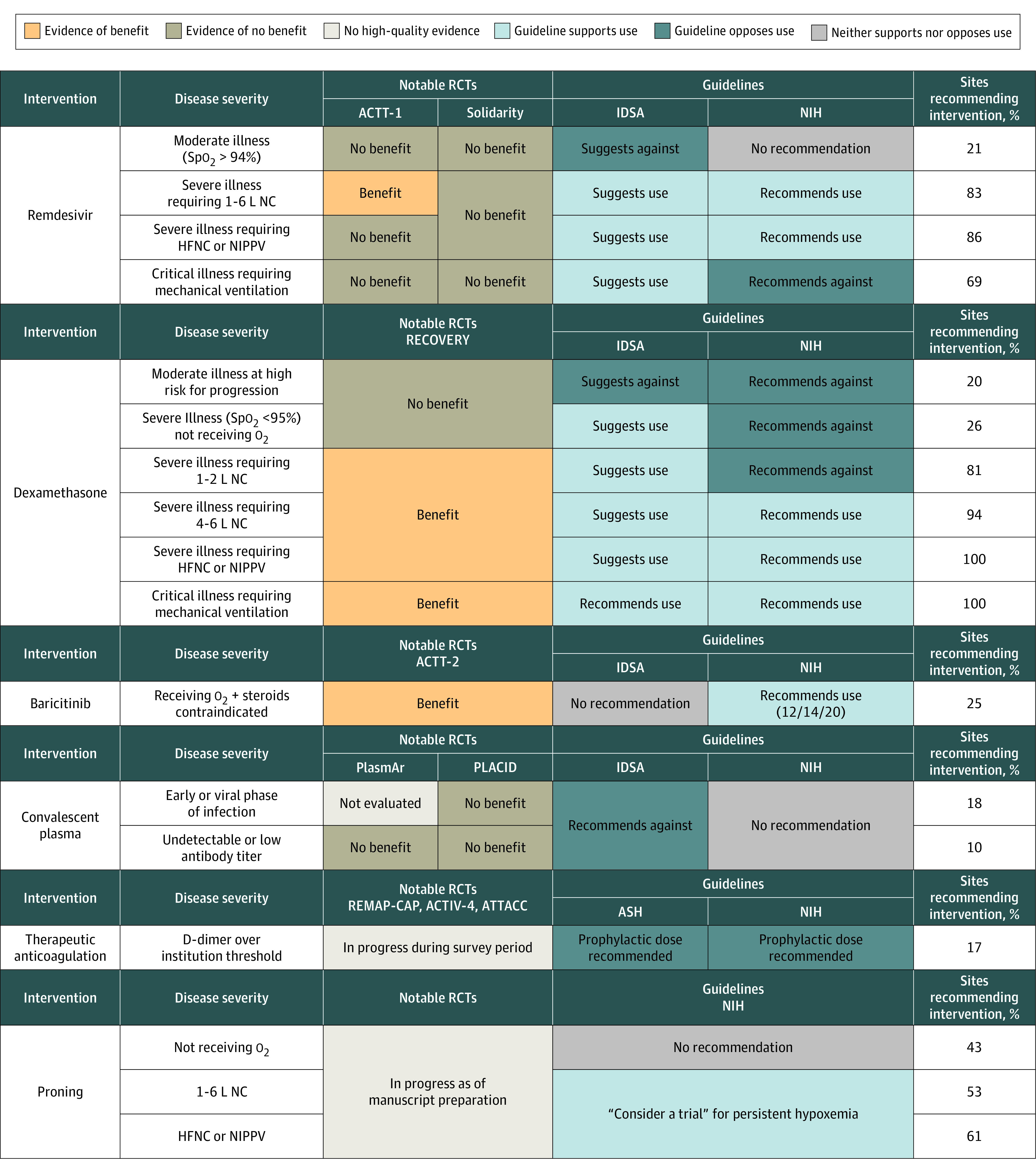
Comparing Academic Medical Centers’ Institutional Recommendations to National Guidelines and Randomized Clinical Trial (RCT) Data The percentage of sites recommending use of each clinical intervention for a given patient population, stratified by disease severity, is presented alongside simplified findings from notable RCTs (eAppendix in the Supplement) and guidelines available at the time of the survey from the National Institutes of Health (NIH),^2^ Infectious Diseases Society of America (IDSA),^3^ and American Society of Hematology (ASH).^4^ No published RCTs were available at the time of the survey for anticoagulation or proning. ACTIV-4 indicates Anti-thrombotics for Adults Hospitalized With COVID-19; ACTT, Adaptive COVID-19 Treatment Trial; ATTACC, Antithrombotic Therapy to Ameliorate Complications of COVID-19; HFNC, high-flow nasal cannula; NC, nasal cannula; NIPPV, noninvasive positive-pressure ventilation; PLACID, Convalescent Plasma in the Management of Moderate COVID-19 in Adults in India; PlasmAr, Convalescent Plasma and Placebo for the Treatment of COVID-19 Severe Pneumonia; RECOVERY, Randomized Evaluation of COVID-19 Therapy; REMAP-CAP, A Randomised, Embedded, Multi-factorial, Adaptive Platform Trial for Community-Acquired Pneumonia; Spo_2_, oxygen saturation as measured by pulse oximetry.

Of 83 hospitals contacted, 52 (63%) responded. Hospitalist leaders involved in the direct care of patients with COVID-19 provided responses. A total of 49 sites (94%) self-identified as AMCs; the remaining 3 sites (6%) identified as AMC-affiliated teaching hospitals. Fifty-one sites (98%) issued internal COVID-19 management guidance. Guidance at 48 sites (94%) was generated by multidisciplinary committees, including infectious disease (47 sites [98%]), infection control (43 sites [90%]), hospital medicine (42 sites [88%]), and critical care (40 sites [83%]). Of 51 sites with internal COVID-19 management guidance, recommendations were disseminated most commonly through email (43 sites [84%]), institutional websites (42 sites [82%]), and integration into the electronic health record as COVID-19-specific order sets (37 sites [73%]) and note templates (33 sites [65%]). The percentage of institutions recommending each studied intervention is shown in Figure 2, alongside simplified RCT findings and guidelines. Notable results include 94% to 100% of sites recommending dexamethasone for patients requiring at least 4 L of oxygen, 69% recommending remdesivir for patients receiving mechanical ventilation, 81% recommending dexamethasone for patients requiring 1 to 2 L of oxygen, 67% implementing awake proning, 35% limiting remdesivir use to the “early or viral phase of illness,” and 17% recommending D-dimer–based therapeutic anticoagulation. The proportion of sites recommending each intervention varied from a low of 10% for convalescent plasma for patients without antibodies to 100% for dexamethasone in patients requiring mechanical ventilation.

## Discussion

In this survey study, 3 themes emerged from our analysis. First, translation from evidence to practice guidelines was remarkably complete for interventions supported by aligned national guidelines and high-quality studies. A striking example is the near universal adoption of dexamethasone among patients requiring at least 4 L of supplementary oxygen only 6 to 8 months after the RECOVERY trial demonstrated a survival benefit. The lone exception to this trend was baricitinib; however, new evidence and guidelines were released within 1 week of survey distribution. Practice convergence was also observed when evidence and guidelines aligned against interventions, as seen in the infrequent recommendation of dexamethasone for patients with oxygen saturation greater than 94%, as measured by pulse oximetry. However, clear opportunity for improvement still exists.

Second, institutions favored treatment over not treatment, particularly when guidelines diverged from each other or from the underlying evidence, as exemplified by 69% to 81% of sites recommending remdesivir or dexamethasone, respectively, when evidence or guidelines conflicted. We suspect this finding reflects systemic biases to do something rather than nothing when faced with uncertainty, likely exacerbated by inconsistent definitions of disease severity across studies and guidelines.

Finally, AMCs demonstrated a willingness to innovate across a range of interventions. Novel interventions, such as awake proning, phase-of-illness–restricted remdesivir, and D-dimer–based therapeutic anticoagulation, varied widely with respect to patient selection and procedural specifics, but collectively, and with 17% to 67% of sites recommending 1 or more of such novel interventions, they demonstrate that AMCs were sophisticated consumers of information, willing to bridge knowledge gaps with expert opinion. Additional research is needed to understand how AMCs monitor innovation outcomes and deimplement practices when negative evidence emerges.

Limitations of this study include a 37% nonresponse rate, reliance on self-reporting, lack of longitudinal follow-up, and lack of data on actual clinical practice and outcomes. Nonetheless, our findings demonstrate that AMCs were capable of responding nimbly to emerging data and shifting guidelines, although both overtreatment and experimentation were observed where significant uncertainty persisted. While factors unique to the early pandemic likely shaped this performance, we hope some strategies, such as use of focused multidisciplinary teams and novel information sharing tools, can be harnessed to accelerate the translation of evidence to bedside for COVID-19 and beyond.

## Notes

Supplement. **eAppendix.** Pivotal Randomized Clinical Trials
