# Supplementary material for: Implementation of Clinical Practice Guidelines for Hospitalized Patients With COVID-19 in Academic Medical Centers
Source: JAMA Netw Open. 2022 Apr 4;5(4):e225657. doi: 10.1001/jamanetworkopen.2022.5657 (PMC8980917; doi:10.1001/jamanetworkopen.2022.5657)
Supplement: Supplement. — eAppendix. Pivotal Randomized Clinical Trials [file jamanetwopen-e225657-s001.pdf]

Supplemental Online Content

Berger AC, Simchoni N, Auerbach A, et al; HOMERuN COVID-19 Collaborative Group. Implementation of clinical practice guidelines for hospitalized patients with COVID-19 in academic medical centers. *JAMA Netw Open*. 2022;5(4):e225657. doi:10.1001/jamanetworkopen.2022.5657

eAppendix. Pivotal Randomized Clinical Trials

This supplemental material has been provided by the authors to give readers additional information about their work.

© 2022 Berger AC et al. *JAMA Network Open.*

eAppendix. Pivotal Randomized Clinical Trials

Remdesivir:

A. Beigel JH, Tomashek KM, Dodd LE, et al. Remdesivir for the Treatment of Covid-19 - Final Report. N Engl J Med. 2020;383(19):1813-1826. doi:10.1056/NEJMoa2007764

B. WHO Solidarity Trial Consortium, Pan H, Peto R, et al. Repurposed Antiviral Drugs for Covid-19 - Interim WHO Solidarity Trial Results. N Engl J Med. 2021;384(6):497-511. doi:10.1056/NEJMoa2023184.

Dexamethasone:

RECOVERY Collaborative Group, Horby P, Lim WS, et al. Dexamethasone in Hospitalized Patients with Covid-19. N Engl J Med. 2021;384(8):693-704. doi:10.1056/NEJMoa2021436.

Baricitinib:

Kalil AC, Patterson TF, Mehta AK, et al. Baricitinib plus Remdesivir for Hospitalized Adults with Covid-19. N Engl J Med. 2021;384(9):795-807. doi:10.1056/NEJMoa2031994.

Convalescent plasma:

A. Li L, Zhang W, Hu Y, et al. Effect of Convalescent Plasma Therapy on Time to Clinical Improvement in Patients With Severe and Life-threatening COVID-19: A Randomized Clinical Trial [published correction appears in JAMA. 2020;324(5):519]. JAMA. 2020;324(5):460-470. doi:10.1001/jama.2020.10044.

B. Agarwal A, Mukherjee A, Kumar G, et al. Convalescent plasma in the management of moderate covid-19 in adults in India: open label phase II multicentre randomised controlled trial (PLACID Trial) [published correction appears in BMJ. 2020;371:m3939. doi:10.1136/bmj.m3939

© 2022 Berger AC et al. *JAMA Network Open.*
